# Acetylation of NDPK-D Regulates Its Subcellular Localization and Cell Survival

**DOI:** 10.1371/journal.pone.0139616

**Published:** 2015-10-01

**Authors:** Yuki Fujita, Kei Fujiwara, Shigetake Zenitani, Toshihide Yamashita

**Affiliations:** 1 Department of Molecular Neuroscience, Graduate School of Medicine, Osaka University, 2–2 Yamadaoka, Suita, Osaka, Japan; 2 Core Research for Evolutional Science and Technology, Japan Science and Technology Agency, 5, Sanbancho, Chiyoda-ku, Tokyo, Japan; Northwestern University Feinberg School of Medicine, UNITED STATES

## Abstract

Nucleoside diphosphate kinases (NDPK) are ubiquitous enzymes that catalyze the reversible phosphotransfer of γ-phosphates between di- and triphosphonucleosides. NDPK-D (Nm23-H4) is the only member of the NDPK family with a mitochondrial targeting sequence. Despite the high expression of NDPK-D in the developing central nervous system, its function remains to be determined. In this study, we show that NDPK-D knockdown induces apoptosis in neuroblastoma cells as well as in mouse cortex, suggesting that NDPK-D is required for neuronal survival. We identified NDPK-D as a binding partner of NAD^+^-dependent histone deacetylase, SIRT1, by yeast two-hybrid screening. NDPK-D co-localized with SIRT1, and the association of these molecules was confirmed by co-immunoprecipitation. Inhibition of SIRT1 increases the acetylation of NDPK-D. Overexpression of NDPK-D along with SIRT1, or mutation in the acetylated lysine residues in NDPK-D, increases its nuclear accumulation. Furthermore, the NDPK-D acetylation-mimic mutant increased apoptosis in N1E-115 cells. Our data demonstrate that acetylation regulates the shuttling of NDPK-D between nucleus and cytoplasm, and increased acetylation of NDPK-D causes apoptosis.

## Introduction

The sirtuins are a conserved class of proteins, which possess NAD^+^-dependent deacetylase and adenosine diphosphate (ADP)-ribosyl transferase activity [1–5]. Originally identified in yeast, silent information regulation 2 (Sir2) [6, 7] regulates life span by inhibiting genomic instability via chromatin modification. In mammals, seven sirtuin homologs (SIRT1-7) identified are categorized into four classes based on their DNA sequence. SIRT1, the mammalian homolog of Sir2 belongs to class I sirtuins [8]. Because the activity of SIRT1 depends on NAD^+^, the energy status of the cell and nutrient deprivation such as fasting and caloric restriction may affect its function [9].

SIRT1 mediates diverse aspects of cells, including survival, differentiation, and metabolism, through the deacetylation of target molecules [10, 11]. The perinatal death of SIRT1 knockout mice appears to be associated with growth retardation and developmental abnormalities of eye, lung, and heart, suggesting that SIRT1 is crucial to development [12, 13]. Moreover, SIRT1 inhibits neurodegeneration in *in vivo* and *in vitro* models of Alzheimer's disease, amyotrophic lateral sclerosis (ALS), and Wallerian degeneration [14–17]. Thus, SIRT1 is important for neuronal protection against neurotoxic insults. SIRT1 is also involved in learning and memory formation as SIRT1 deficient mice show decreased dendritic complexity and impaired hippocampal-dependent memory [18, 19]. SIRT1 deficiency increases brain-specific miR-134 expression, leading to decreased expression of cAMP response element-binding protein (CREB) and brain-derived neurotrophic factor (BDNF), and this results in impaired synaptic plasticity [20]. SIRT1 agonist resveratrol suppresses miR-134 and miR-124, which increase CREB and BDNF expression [21]. These studies clearly indicate that SIRT1 is vital to the development of central nervous system.

In addition to modulating gene silencing by repressive heterochromatin formation via histone deacetylation, SIRT1 targets diverse factors such as FOXO, p53, NF-κB, PPAR-gamma co-activator 1-alpha (PGC-1α) and peroxisome proliferator-activated receptor gamma (PPARγ) [22–27]. In particular, SIRT1 and PGC-1α loicalize within mitochondria, and regulate mitochondrial biogenesis and metabolism by mediating the cross talk between nuclear and mitochondrial genome [28]. The localization of SIRT1 in the nuclear and mitochondrial fraction further supports its role as a crucial regulator of mitochondrial function [28–31].

We previously screened for the binding partner of SIRT1 using the yeast two-hybrid system on human fetal brain cDNA library [32]. We found that nucleoside diphosphate kinases D (NDPK-D/Nm23-H4) encoded by the Nme4 (nm23-H4) gene [33], is a novel SIRT1 binding partner. The human Nm23 family comprises ten members (Nm23-H1 to H10, H means the human isoform). These proteins are distributed into two groups based on their gene sequence and catalytic activity [34]. Group I proteins (Nm23-H1, H2, H3, and H4) have conserved NDP kinase active site motifs and phosphotransferase activity. Group II proteins (Nm23-H5, H6, H7, and H8) demonstrate high sequence diversity. Nm23-H9 and-H10/RP2, which are novel Nm23 members, seem to belong to Group II, according to motif prediction and phylogenetic data.

NDPK-D (Nm23-H4) is the only NDPK with a specific mitochondrial targeting sequence. NDPK-D forms homohexamers and catalyzes the reversible exchange of γ-phosphate between nucleoside diphosphates and triphosphates [33, 35, 36]. The catalytic reaction of NDPK follows a ping-pong reaction with the transient appearance of a high-energy phosphorylated histidine residue. Mutation of the central arginine (R90D) in a basic RRK motif (R89–R90–K91) that is situated on the surface-exposed loop in each monomer of the hexameric complex interferes with NDPK-D mitochondrial localization [37]. However, physiological function of NDPK-D remains unclear. In this study, we demonstrate that NDPK-D knockdown induces cell death through caspase-3 activation in N1E-115 neuroblastoma cell lines. Cell death was increased by *in utero* electroporation of NDPK-D siRNA in embryonic mouse brain. Furthermore, mutation in the acetylated lysine residues reduced the mitochondrial localization of NDPK-D, suggesting that acetylation regulates the subcellular localization of NDPK-D.

## Materials and Methods

### Antibodies and Reagents

Ex527 and z-VAD-FMK were purchased from Sigma-Aldrich (MO, USA) and R&D systems (MN, USA) respectively. Antibodies used for co-immunoprecipitation, western blotting, and immunostaining were as follows: monoclonal anti-HA (HA-7, Sigma-Aldrich), anti-c-Myc (9E10) and anti-α tubulin (Santa Cruz Biotechnology, CA, USA), anti-Histone H3 (Millipore, MA, USA); polyclonal anti-Myc (Millipore), anti-HA and anti-VDAC (Abcam, Cambridge, MA), anti-GFP (Invitrogen, CA, USA), anti-Iba1 (Wako, Japan), anti-acetylated-Lysine, anti-cleaved caspase-3, and anti-β actin (Cell signaling technology, MA, USA); secondary horseradish peroxidase (HRP)-conjugated anti-mouse and anti-rabbit IgG (Cell signaling Technology) and Alexa 488 or 568 conjugated goat anti-mouse and goat anti-rabbit IgG (Molecular Probes, OR, USA).

### Cell culture and transfection

Human embryonic kidney cell line HEK 293T cells (ATCC CRL-11268) and mouse neuroblastoma N1E-115 cells (ATCC CRL-2263) were cultured in DMEM (Invitrogen) supplemented with 10% fetal bovine serum (FBS) and 50 U penicillin-streptomycin at 37°C with 5% CO_2_ inside a humidified incubator. Transient transfection was performed using Lipofectamine 2000 (Invitrogen) according to the manufacturer's instructions.

### Plasmid constructs and siRNA

cDNA fragments encoding human SIRT1 were PCR amplified from pMyc-SIRT1[32] and sub-cloned into pHA-C1 mammalian expression vector containing the HA epitope tag[38]. cDNA fragments encoding human NDPK-D were amplified by PCR from pCMV-SPORT6-NDPK-D (ATCC Number 6400207) and sub-cloned into the EcoR1-Sal1 site of the pMyc-C1 mammalian expression vector containing the Myc epitope tag. All site-specific mutagenesis experiments were carried out using the KOD-Plus-Mutagenesis Kit (TOYOBO, Shiga, Japan) using pMyc-C1-NDPK-D vector as a template and the following primers: K45R-Forward: 5 ʹ-AGGCCCGATGGCGTGCAACGGCGGC-3 ʹ, K45Q-Forward: 5 ʹ-CAGCCCGATGGCGTGCAACGGCGGC-3 ʹ, K45R/Q-Reverse: 5 ʹ-CACCGCCACCAGGGTCCGCTCCCGG-3 ʹ, K72R-Forward: 5 ʹ-AGGATGCTGCAGGCACCAGAGAGCG-3 ʹ, K72Q-Forward: 5 ʹ-CAGATGCTGCAGGCACCAGAGAGCG-3 ʹ, K72R/Q-Reverse: 5 ʹ-CATCCCCACCAGCGTGAAGCCCCGC-3 ʹ, K91R-Forward: 5 ʹ-AGGCCCTTCTACCCTGCCCTCATCC-3 ʹ, K91Q-Forward: 5 ʹ-CAGCCCTTCTACCCTGCCCTCATCC-3 ʹ, K91R/Q-Reverse: 5 ʹ-CCTCCGCAGGTCCTGGTAGTGCTCG-3 ʹ. Mitochondria-targeted GFP was constructed by ligating subunit VIII of cytochrome C oxidase and GFP.

Stealth siRNA was purchased from Invitrogen. Target sequences were as follows: NDPK-D siRNA #1-sense: GGAAGCCAUUCUACCCAGCUCUUAU, NDPK-D siRNA #1-antisense: AUAAGAGCUGGGUAGAAUGGCUUCC, NDPK-D siRNA #2-sense: GCCAUGAUAGGACACACCGACUCAA, NDPK-D siRNA #2-antisense: UUGAGUCGGUGUGUCCUAUCAUGGC, NDPK-D siRNA #3-sense: GGCGACUUCAGUGUUCACAUCAGCA, NDPK-D siRNA #3-antisense: UGCUGAUGUGAACACUGAAGUCGCC. Negative control siRNA (Ambion, TX, USA) with no sequence similarity to mouse gene was used as a control siRNA. The design of SIRT1 shRNA vector was described by previous work [39]. The non-targeting control shRNA sequence was previously described [40]. The shRNA sequence was sub-cloned into pSuper-GFP-Neo vector according to the manufacturer’s protocol.

#### RNA extraction, reverse transcription, and real-time PCR

Total RNA was extracted from mouse brains at the indicated age with Trizol (Invitrogen) and reverse transcribed using the High-Capacity cDNA Reverse Transcription Kit (Applied Biosystems, CA, USA). The mRNA expression was determined using a 7300 fast real-time PCR system (Applied Biosystems) and SYBR Green assay (vendor) was performed to quantify NDPK-D expression. Primers for mouse NDPK-D were designed by Primer Express software (Applied Biosystems). The sequences were as follows: Forward, 5 ʹ-GGCGACTTCAGTGTTCACATCA-3 ʹ; Reverse, 5 ʹ-GGGCCCCATCCACAGAAT-3 ʹ. The specificity of each primer set was determined with a pre-test confirming the amplification for a specific gene by gel visualization and sequencing. 20 μL was used for SYBR green assays, and it contained a 1X final concentration of Power SYBR green PCR master mix (Applied Biosystems), 400 nM gene-specific primers, and 1 μL template. The PCR cycles were initiated with an UNG digestion stage at 50°C for 2 min, and an initial denaturation period at 95°C lasting for 10 min, followed by 42 cycles at 95°C for 15 s, annealing at 60°C for 1 min, and a gradual increase in temperature from 60 to 95°C during the dissociation stage. The relative mRNA expression was normalized to the amount of GAPDH mRNA in each sample. The results of cycle threshold values (Ct values) were calculated by the ΔΔCt method to obtain the fold differences.

### Co-immunoprecipitation

Co-immunoprecipitation was performed as previously described[41]. Cells were transfected with plasmids encoding Myc-NDPK-D and/or HA-SIRT1 using Lipofectamine 2000 reagent. The transfected cells were cultured for 36 to 48 h. To immunoprecipitate Myc-tagged proteins, the cells were lysed in a lysis buffer containing 50 mM Tris–HCl (pH 7.4), 150 mM NaCl, 0.5–1% NP-40, 10 mM NaF, 1 mM Na_3_VO_4_, and a protease inhibitor cocktail (Roche Diagnostics K.K., Tokyo, Japan). The whole cell lysates were pre-cleared with rProtein G Sepharose Fast Flow (GE Healthcare, Chalfont St Giles, England) at 4°C for 1 h. The supernatants were incubated at 4°C for 4 h with anti-Myc antibody. The immune complexes were collected after incubation for 1 h at 4°C with protein G Sepharose (GE Healthcare). After washing three times in a wash buffer, the immunoprecipitates were boiled in 2X sample buffer for 5 min and subjected to western blotting.

### Western blotting

Nuclear and cytoplasmic extracts were prepared by NE-PER reagents (Thermo Scientific, NH, USA) according to the manufacturer's instructions. The immunoprecipitates and protein extracts were subjected to SDS-PAGE and Western blotting as described previously[42].

### Deacetylation assay

HEK293T cells were transfected with plasmids encoding Myc-NDPK-D. After 48 h culture in the presence or absence of 50 nM Ex527 (SIRT1 inhibitor) and 25 nM trichostatin A (HDAC inhibitor), the cells were lysed with a lysis buffer. Lysates were immunoprecipitated with anti-Myc antibody and the acetylation levels of NDPK-D were detected by anti-acetylated lysine antibody.

### Immunofluorescence

N1E-115 cells transfected with plasmids encoding Myc-NDPK-D and/or HA-SIRT1 were cultured for 36 h. In some experiments, cells were treated with leptomycin B (LMB) during the last 6 h. Then, the cells were fixed with 4% paraformaldehyde for 1 h and rehydrated by PBS. Cells were permeabilized and non-specific sites were blocked by incubating with PBS containing 0.3% Triton X-100 and 5% bovine serum albumin (BSA). The cells were incubated with antibodies diluted (1:1000) in a blocking solution for 1 h at 4°C. Then, the cells were washed in PBS and incubated with fluorescence-conjugated secondary antibodies, for 1 h at room temperature. Subsequently, the slides were mounted with a fluorescent mounting medium (DakoCytomation, Glostrup, Denmark) after 4’,6-diamidino-2-phenyl-indole (DAPI) staining. Images were acquired on a microscope (BX51; Olympus, Tokyo, Japan) equipped with a camera (DP71; Olympus) using the DP Controller software (version 3.1.1.267; Olympus).

### LDH assay

Cytotoxicity was determined by measuring the activity of lactate dehydrogenase (LDH) in the media using LDH cytotoxicity detection kit (Takara Bio, Inc., Shiga, Japan) according to the manufacturer's instructions. N1E-115 cells were treated with DMSO (control) or 50 μM z-VAD-FMK for 1 h. The cells were then transfected with control or mouse NDPK-D siRNA using Lipofectamine 2000. After 8 h incubation, the medium was replaced with 1 mL of fresh medium containing DMSO or z-VAD-FMK. Forty-eight hours post-transfection, 100 μL of medium was collected in a 96-well plate, and LDH activity was measured at 490 nm using a plate reader (SpectraMax M2, Molecular Devices). Medium alone served as a control for background LDH level and media with only cells were used for low control. Cells exposed to 0.1% Triton X-100 were used as a positive control. All samples were run in triplicates. After LDH assay, cells were fixed and immunostained with anti-cleaved caspase-3 antibody as above described.

### *In situ* hybridization

The cDNA fragments of mouse NDPK-D were amplified by RT-PCR using the in situ hybridization primers previously described[43]. The amplified fragments were TA-cloned into the pGEM-T Easy vector (Promega, WI, USA). Serial sagittal sections, 16 μM thick were mounted on APS-coated glass slides (Matsunami Glass, Osaka, Japan) and processed for in situ hybridization as described previously[44].

### Ethics Statement

The Animal Research Committee of the Osaka University Graduate School of Medicine approved this study. All experiments were performed in accordance with the Guideline for animal experimentation from the Animal Research Committee of the Osaka University Graduate School of Medicine (Permit Number: 24-067-005). All *in vivo* studies were performed under anesthesia.

### *In utero* electroporation

We obtained seven C57BL/6J mice (E14) from Japan SLC, Inc. (Shizuoka, Japan). These mice were maintained at the specific pathogen-free animal room in the Institute of Experimental Animal Sciences, Osaka University Graduate School of Medicine. Mice were housed one per cage after *in utero* electroporation, in a room with a 12-h light/dark cycle with access to food and water ad libitum. Mice were anesthetized and a midline incision was performed to access the embryos. Non-targeting control siRNA or NDPK-D siRNA #1 (100 pmol) along with 0.5 μg of pEGFP-C1 (Clontech, CA, USA) reporter plasmid were injected into the lateral ventricles. After injection, electroporation (five pulses of 40 V; duration, 50 msec; interval, 950 msec) was performed using CUY21 (Nepagene, Japan). After the electroporation, mice were warmed with a heating pad until they recovered from the anesthesia. After 3 days, embryos were harvested and fixed in 4% paraformaldehyde (PFA) overnight at 4°C. The brains (control siRNA, n = 5; NDPK-D siRNA #1, n = 5) were cryoprotected in 10–30% sucrose overnight at 4°C and embedded in OCT compound (Tissue Tek). Brains were frozen in dry ice and serial cross sections (25 μm) were prepared using a cryostat and collected on MAS-coated glass slides. The slides were examined by two masked people, and loss of brain cells was assessed as positive or negative.

### Immunohistochemistry

Brain sections were incubated with blocking solution containing 5% BSA and 0.1% Triton X-100 in PBS for 1 h at room temperature, followed by overnight incubation with the indicated antibodies at 4°C. Immunoreactivity was visualized using fluorescence-conjugated secondary antibodies. Coverslips were then mounted with mounting medium after 1 μg/mL 4ʹ,6-diamidino-2ʹ-phenylindole dihydrochloride (DAPI) staining.

## Results

### Expression profile of NDPK-D in the brain

**Fig 1:**
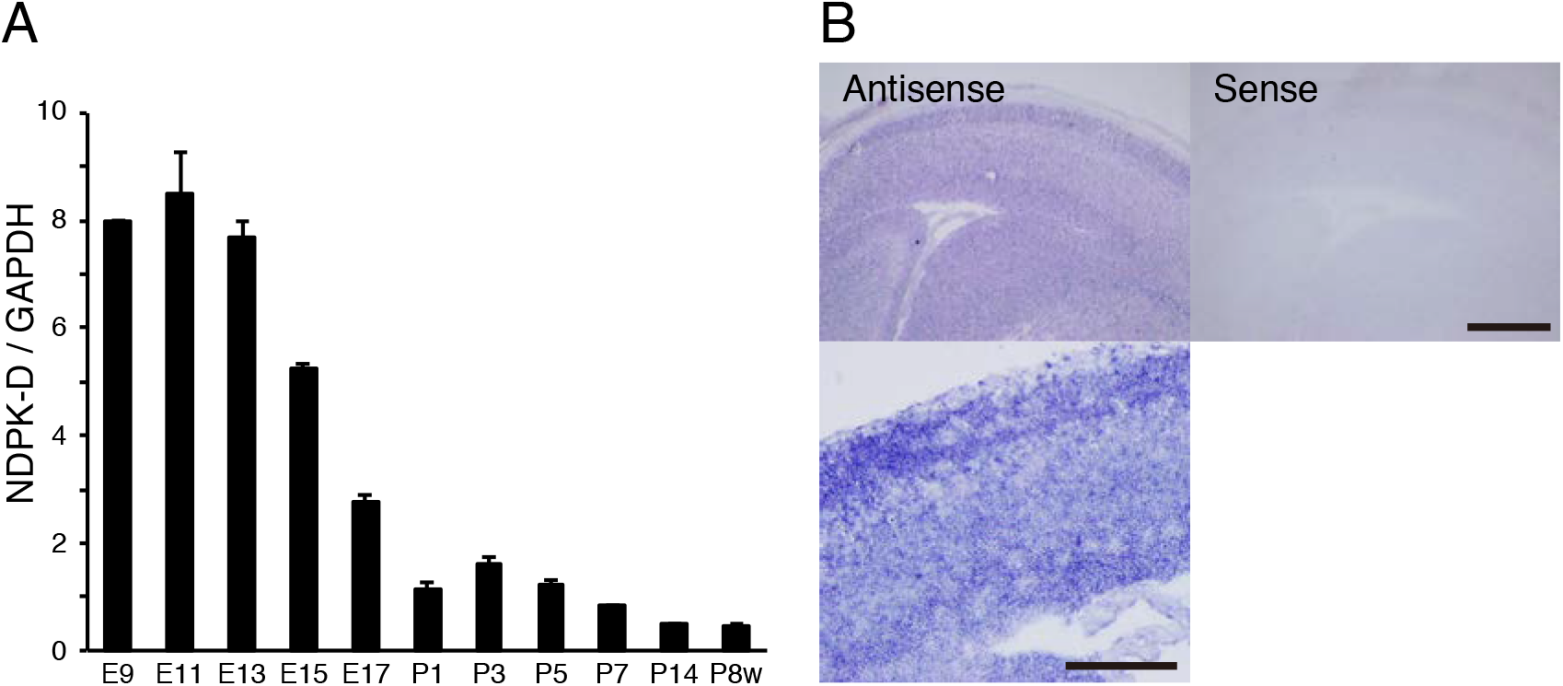
Relative NDPK-D expression patterns in mouse brain at different developmental stages. (A) Expression profiles of mouse cDNA from cerebrum at various developmental stages. The expression levels of NDPK-D relative to those of GAPDH were measured by ΔΔCt method. The results were the mean ± SE from three independent experiments. The relative NDPK-D expression levels are presented as fold changes relative to the level in P1. (B) In situ hybridization of NDPK-D mRNA in sagittal section of E15 mouse brain. Expression of NDPK-D mRNA was detected in both cortical plate and ventricular zone. Scale bar: 500 μm (low magnification image), 200 μm (high magnification image).

We first examined the NDPK-D expression profile by real-time PCR, using total RNA extracted from mouse brains at various developmental stages. NDPK-D expression was relatively higher in the embryonic stage than in the adult stage (Fig 1A). Next, we investigated the distribution of NDPK-D in the brain by *in situ* hybridization. Sagittal sections of mouse brain at E15 were prepared and hybridized with NDPK-D antisense or sense probe. Hybridization with NDPK-D antisense RNA probe displayed widespread expression, with relatively higher expression of NDPK-D mRNA in several regions including ventricular zone and cortical plate (Fig 1B). The absence of hybridization signals with sense control probe eliminated any background noise and confirmed specific NDPK-D mRNA expression in the brain tissues (Fig 1B).

### Knockdown of NDPK-D induces apoptosis

**Fig 2:**
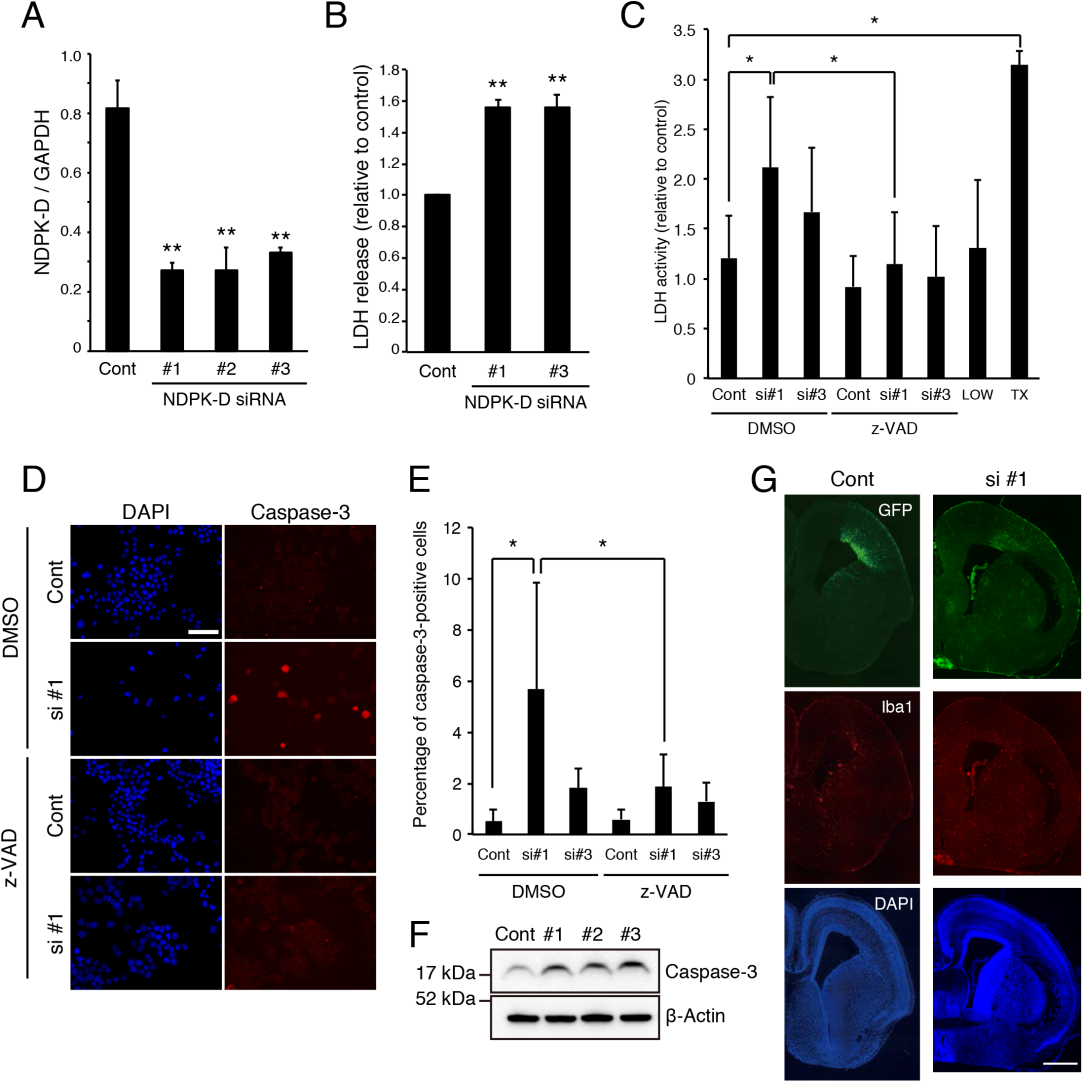
NDPK-D Knockdown induces apoptosis of N1E-115 cells. (A) NDPK-D siRNAs reduced NDPK-D mRNA expression. N1E-115 cells were transfected with the indicated siRNAs. Total RNA isolated at 72 h post-transfection was analyzed by real-time PCR. **P < 0.01. n = 3. (B) siRNA-mediated knockdown of NDPK-D increased LDH release. N1E-115 cells were transfected with indicated siRNA and cultured for 48 h. The relative LDH activities were measured in the culture medium and normalized with control values. **P < 0.01. n = 3. (C) z-VAD-FMK rescues NDPK-D siRNA induced apoptosis. N1E-115 cells were transfected with indicated siRNA and treated with or without 50 μM z-VAD-fmk. Cells were cultured for 48 h and LDH activities were measured as described in (B). LOW: no transfection; TX: 0.1% triton-X 100; *P < 0.05. n = 3. (D, E) siRNA-mediated NDPK-D knockdown increased the number of cleaved caspase-3-positive cells. N1E-115 cells transfected with indicated siRNAs were immunostained with anti-cleaved caspase-3 antibody. The representative images of transfected N1E-115 were shown (D). Percentage of cleaved caspase-3-positive cells were demonstrated in the graph (E). *P < 0.05. Scale bar: 100 μm. n = 3. (F) NDPK-D knockdown produces cleaved caspase-3. N1E-115 cells were transfected with indicated siRNAs. Cell lysates were prepared 72 h after transfection and subjected to western blotting. Cont: control siRNA; si #1, 2, 3, NDPK-D siRNA #1, 2, 3. (G) Knockdown of NDPK-D increased cell death in mouse embryo. Representative images of E17 brain sections from embryos that were co-transfected with GFP and NDPK-D siRNA #1 at E14. The sections were immunostained with anti-GFP and anti-Iba1 antibodies. Scale bar: 600 μm. Statistical analyses were performed using one-way ANOVA followed by Scheffe’s (A, B), or Tukey-Kramer’s (C, E) multiple comparison tests.

To investigate the physiological function of NDPK-D, we performed loss-of-function experiments using small interfering RNA (siRNA). We first examined the knockdown efficacy of NDPK-D siRNA in neuroblastoma N1E-115 cells. Efficient NDPK-D mRNA downregulation specifically occurred in NDPK-D siRNA-transfected, but not in the control, non-target siRNA-transfected cells (Fig 2A). We then examined whether NDPK-D knockdown regulated cellular viability by the lactate dehydrogenase (LDH) activity assay. N1E-115 cells were transfected with control or NDPK-D siRNA and cultured for 48 h. LDH release was greater in NDPK-D siRNA-transfected cells than in the control siRNA-transfected cells (Fig 2B). Moreover, pan-caspase inhibitor, z-VAD-FMK suppressed the release of LDH from the cells (Fig 2C). In addition, immunostaining demonstrated a significant increase (*P* < 0.05) in cleaved caspase-3-positive cells in the presence of NDPK-D siRNA (Fig 2D). z-VAD-FMK significantly inhibited (*P* < 0.05) the NDPK-D siRNA-induced caspase activation (Fig 2E). Western blot analysis further confirms that NDPK-D downregulation induces caspase-3 activation (Fig 2F). Taken together, these results indicate that knockdown of NDPK-D increases apoptosis through caspase-3 activation in N1E-115 cells. Then, to assess the effect of NDPK-D inhibition *in vivo*, we performed *in utero* electroporation. Embryonic day (E) 14 mouse embryos were co-transfected with GFP and NDPK-D siRNA #1. Embryos were fixed and analyzed 3 days after the *in utero* electroporation. Knockdown of NDPK-D increased the accumulation of Iba1-positive microglia (Fig 2G), suggesting phagocytosis of dead cells by microglia. These results support the notion that NDPK-D is required for neuronal survival both *in vitro* and *in vivo*.

### Interaction of NDPK-D with SIRT1

**Fig 3:**
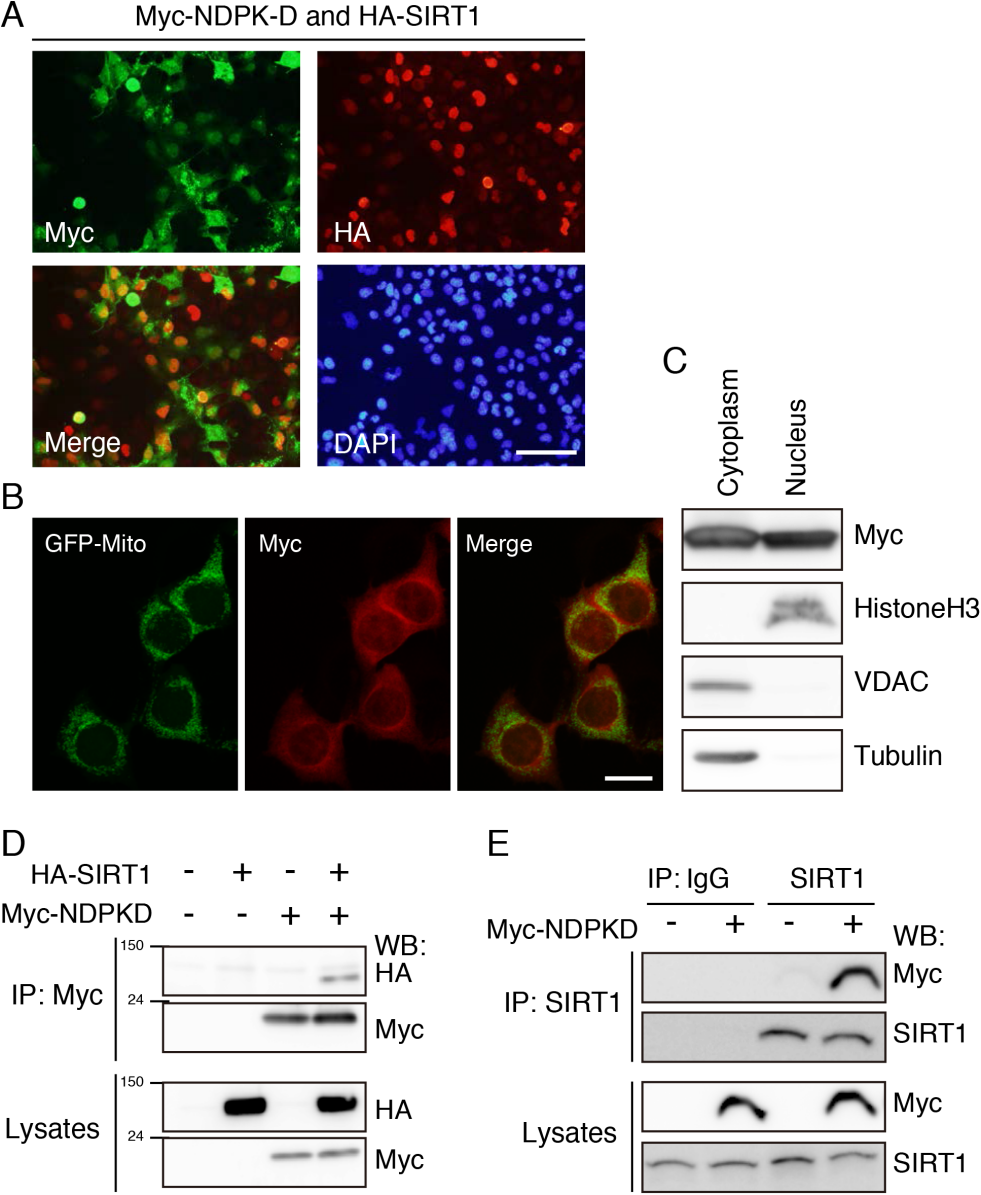
SIRT1 interacts with NDPK-D. (A) Localization of SIRT1 and NDPK-D. COS-7 cells transfected with plasmids encoding HA-SIRT1 and Myc-NDPK-D were cultured for 36 h, and immunostained with anti-HA and anti-Myc antibodies. Scale bar: 100 μm. (B) Cytosolic NDPK-D partially co-localized with mitochondria-targeted GFP. Cells transfected with plasmids encoding Myc-NDPK-D and mitochondria-targeted GFP were immunostained with anti-Myc and anti-GFP antibodies. Scale bar: 20 μm. (C) NDPK-D localized to both cytoplasmic and nuclear fractions. COS-7 cells were transfected with Myc-NDPK-D, and cultured for 36 h. Cytoplasmic and nuclear fractions were isolated and subjected to western blotting using indicated antibodies. (D) Co-immunoprecipitation of SIRT1 and NDPK-D. Cells were transiently transfected with the indicated plasmids and lysates were immunoprecipitated with anti-Myc antibody. The immunoprecipitates were immunoblotted with anti-HA antibody. (E) Co-immunoprecipitation of Endogenous SIRT1 and Myc-tagged NDPK-D. Cells were immunoprecipitated with anti-SIRT1 or control IgG antibody. The immunoprecipitates were immunoblotted with anti-Myc antibody.

We previously conducted yeast two-hybrid screen with human fetal brain cDNA library using the conserved sirtuin catalytic domain as the bait [32]. Among the possible SIRT1 binding partners, we focused on human NDPK-D because of its role in mitochondrial biogenesis. To validate the association of SIRT1 with NDPK-D in mammalian cells, we first examined the subcellular localization of SIRT1 and NDPK-D in COS-7 cells. The cells were co-transfected with expression vector carrying Myc-tagged NDPK-D and HA-tagged SIRT1, and immunostained with antibodies against Myc and HA. While SIRT1 predominantly localized to nucleus (Fig 3A), NDPK-D showed mostly cytosol localization with certain variations. NDPK-D was mainly localized in the cytosol with puncta in some cells, and in other cells, was partially localized in the nucleus with diffuse staining in the cytosol (Fig 3A). The punctate NDPK-D signals partially merged with mitochondria-targeted GFP, suggesting that NDPK-D localized to mitochondria (Fig 3B). Western blot analysis on the cytoplasmic and the nuclear lysates were prepared from Myc-NDPK-D transfected COS-7 cells detected NDPK-D protein in both cytoplasmic and nuclear fractions (Fig 3C). These results suggest that NDPK-D localizes to both cytosol and nucleus and that it shares the same subcellular compartments when co-expressed with SIRT1.

To determine the interaction of NDPK-D and SIRT1, COS-7 cells were transiently co-transfected with plasmids expressing Myc-tagged NDPK-D and/or HA-tagged SIRT1. Subsequently, cell lysates were immunoprecipitated with Myc antibody, and SIRT1 was detected using HA antibody (Fig 3D), indicating that ectopically expressed NDPK-D interacts with SIRT1 in COS-7 cells. Based on these data, it appears that binding of NDPK-D to SIRT1 results in their colocalization in COS-7 cells (Fig 3A and 3C). Further, we confirmed the interaction between endogenous SIRT1 and Myc-tagged NDPK-D using N1E-115 cells expressing SIRT1 endogenously. The cells were transfected with Myc-tagged NDPK-D and cell lysates were immunoprecipitated with anti-SIRT1 antibody or control IgG, followed by immunoblot with anti-Myc antibody. Myc-tagged NDPK-D was detected only in the immunoprecipitates with anti-SIRT1 antibody (Fig 3E). Taken together, these results suggest that NDPK-D interacts with SIRT1.

### SIRT1 deacetylates NDPK-D

**Fig 4:**
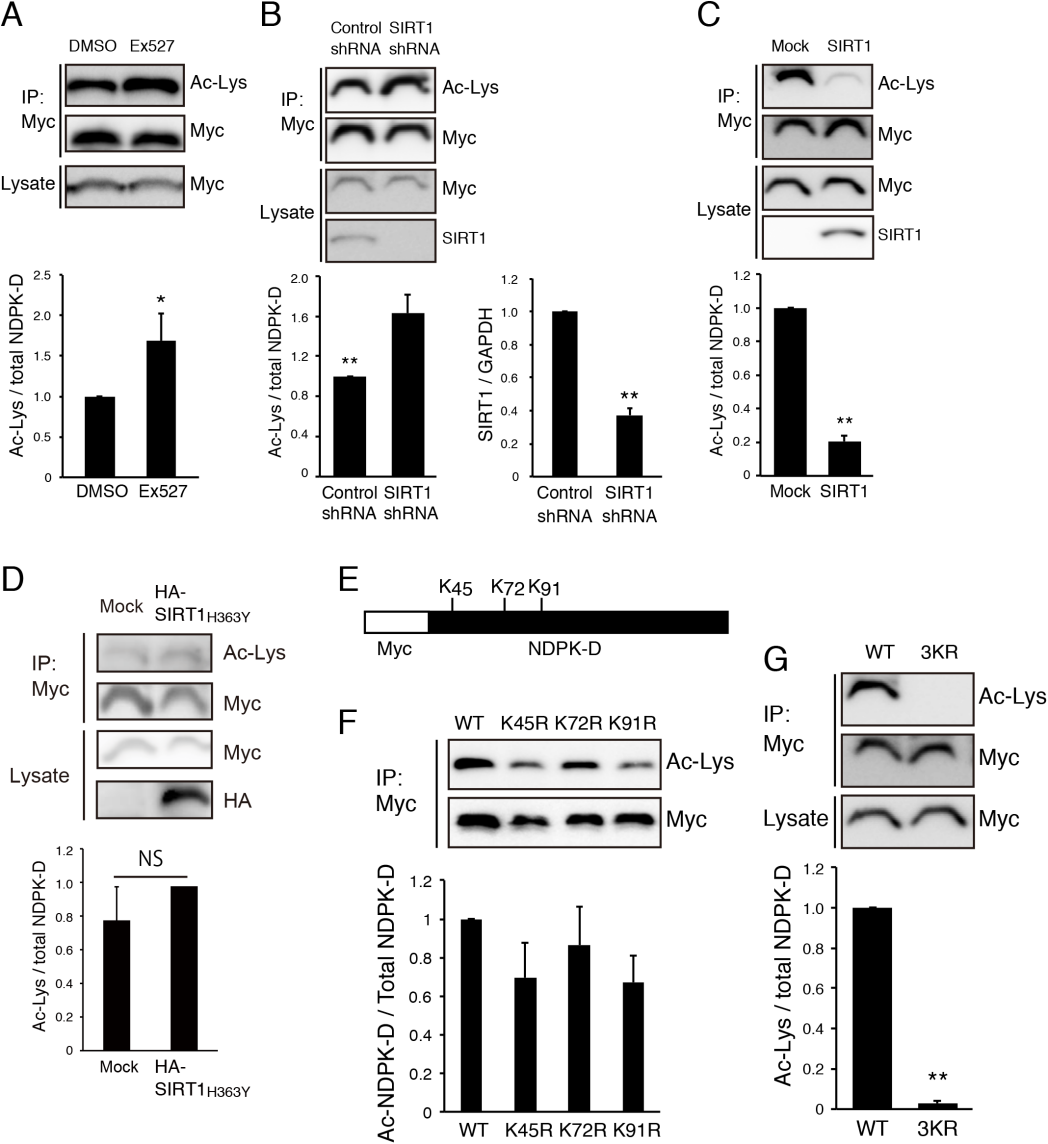
Mutation of acetylated lysine residues results in mislocalization of NDPK-D. (A) Inhibition of SIRT1 increased acetylation level of NDPK-D. N1E-115 cells were transfected with Myc-NDPK-D, treated with SIRT1 inhibitor Ex527, and cultured for 48 h. Cell lysates were immunoprecipitated with anti-Myc antibody and the immunoprecipitates were immunoblotted with anti-acetylated lysine (Ac-Lys) antibody. The Ac-Lys signal intensity was quantified by densitometry and normalized to the signal intensity of precipitated Myc-NDPK-D. *P < 0.05. n = 3. (B) Knockdown of SIRT1 increased acetylation level of NDPK-D. N1E-115 cells were transfected with Myc-NDPK-D and control or SIRT1 shRNA. The acetylation level of NDPK-D was tested as described in (A). **P < 0.01. n = 6. SIRT1 shRNA efficiently reduced Sirt1 mRNA expression in N1E-115 cells (right panel). **P < 0.01. n = 3. (C, D) SIRT1, but not catalytic-dead point mutant (H363Y) deacetylates NDPK-D in N1E-115 cells. Cells were transfected with Myc-NDPK-D and HA-SIRT1 (C) or HA-SIRT1 H363Y (D), and the acetylation levels of Myc-NDPK-D were determined by anti-Ac-Lys antibody. NS: not significant. **P < 0.01. n = 5 (C), n = 3 (D). (E) Schematic representation of lysine residues in NDPK-D. Lys-45, Lys-72, and Lys-91 were candidate acetylation sites. (F) N1E-115 cells were transfected with Myc-tagged wild-type NDPK-D, the K45R, K72R, or K91R mutants. Acetylation levels were determined as described in (A). Replacement of lysine residues with arginine decreased acetylation levels. n = 3. (G) N1E-115 cells were transfected with Myc-tagged wild-type (WT) NDPK-D or the K45/72/91R (3KR) mutant. Acetylation levels were determined as described in (A). **P < 0.01. n = 6. Statistical analyses were performed using Welch’s t-test (A-D, G).

SIRT1 mediated NAD^+^-dependent deacetylation regulates the function of multiple target proteins. Since we employed the SIRT1 deacetylation domain as bait in the yeast two-hybrid screen, it is possible that SIRT1 modulates NDPK-D activity by deacetylation. To test this hypothesis, we performed deacetylation assay. N1E-115 cells were transfected with expression vector encoding Myc-tagged NDPK-D and incubated with or without SIRT1 inhibitor Ex527. NDPK-D was immunoprecipitated with anti-Myc antibody and subjected to western blot analysis using anti-acetylated lysine antibody to examine the acetylation levels of NDPK-D. Elevated levels of acetylated Myc-NDPK-D were detected in the presence of Ex527 relative to the signals in the absence of Ex527, showing that SIRT1 can deacetylate NDPK-D (Fig 4A). We also examined the effect of knockdown of SIRT1 on the acetylation level of NDPK-D. Efficient downregulation of Sirt1 mRNA was found in SIRT1 shRNA-transfected N1E-115 cells (Fig 4B, right graph). The signal for acetylated Myc-NDPK-D increased in SIRT1 shRNA-transfected cells (Fig 4B, left panel and graph). We next examined whether overexpression of SIRT1 deacetylates NDPK-D. N1E-115 cells were transfected with Myc-tagged NDPK-D and HA-tagged SIRT1. The acetylation level of NDPK-D was investigated as described above. The signal for acetylated Myc-NDPK-D was decreased in the presence of HA-tagged SIRT1 (Fig 4C), whereas deacetylase-deficient mutant of SIRT1 (H363Y) did not affect the acetylation level of Myc-NDPK-D (Fig 4D). These results indicate that SIRT1 can modulate the deacetylation level of NDPK-D.

To determine exact acetylated lysine residue (s), we generated NDPK-D mutants in which three lysine residue of NDPK-D (K45, K72, and K91) were replaced by arginine (Fig 4E and S1 Fig). The lysine to arginine (K>R) substitution retains a positive charge and is often used as a deacetylated mimetic form [45–47]. Following overexpression of myc-tagged wild-type NDPK-D, K45R, K72R, and K91R mutants in N1E-115 cells, NDPK-D acetylation levels were evaluated. Each mutant showed reduced acetylation (Fig 4F). Further, we generated an NDPK-D mutant in which all three lysine residues of NDPK-D were replaced by arginine (3KR). The signal for acetylated NDPK-D was significantly lower in 3KR than in the wild type (WT) (Fig 4G). These results suggest that all lysine residues are important for acetylation of NDPK-D.

### Acetylation level of NDPK-D determines its subcellular localization and cellular survival

**Fig 5:**
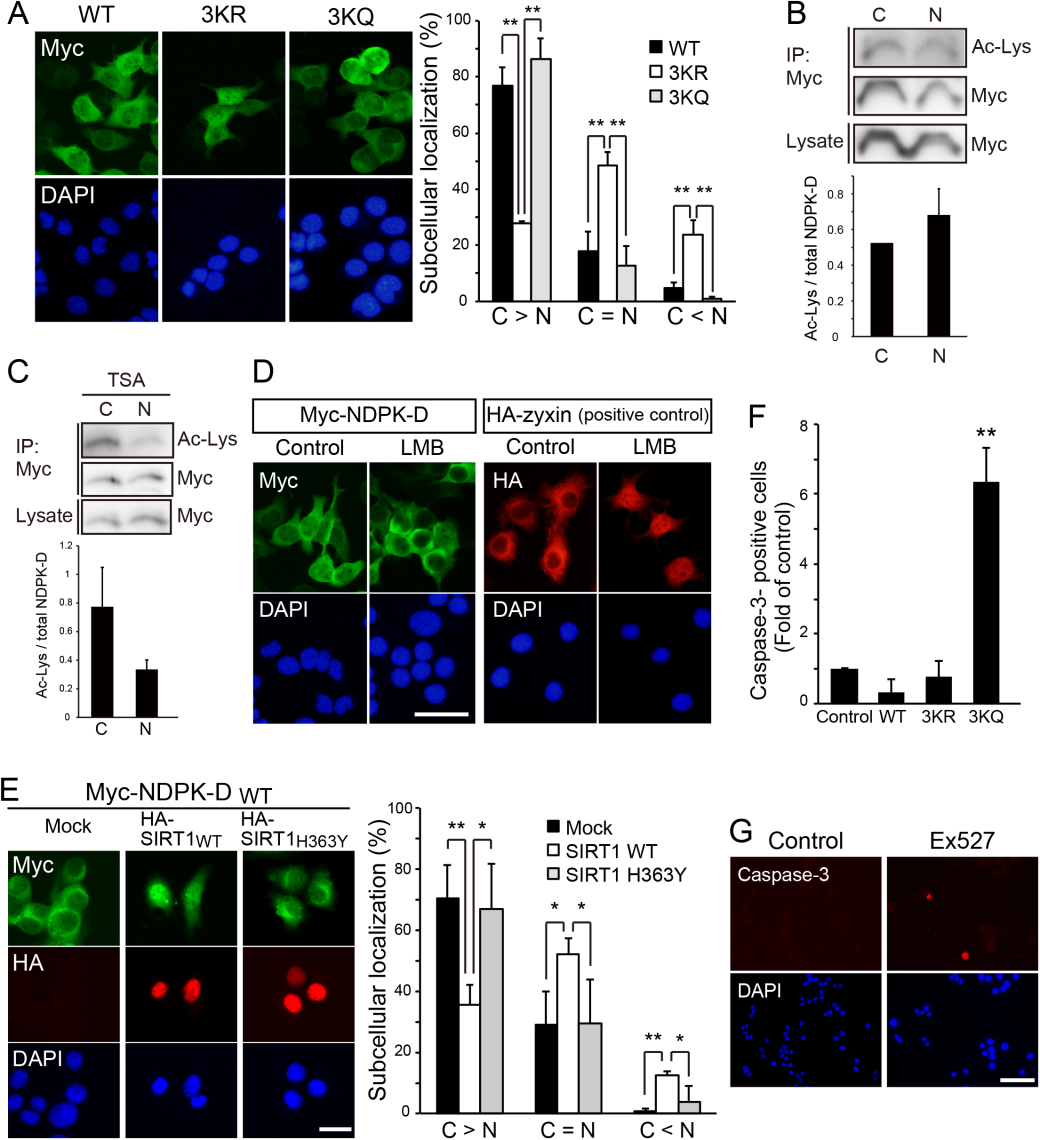
Deacetylation of NDPK-D promoted its nuclear localization. (A) N1E-115 cells were transfected with Myc-tagged wild-type NDPK-D, the 3KR or the K45/72/91Q (3KQ) mutant. WT and 3KQ NDPK-D localized in both cytoplasm and nucleus, whereas the majority of 3KR mutants were retained in the nucleus. NDPK-D localization was classified in the transfected N1E cells. “C > N” indicates the cells predominantly exhibiting Myc-NDPK-D in the cytosol. “C = N” indicates the cells exhibiting cytosol and nuclear Myc-NDPK-D. “C < N” indicates the cells showing nuclear-accumulated Myc-NDPK-D. The percentage of transfected cells exhibiting indicated subcellular localization for NDPK-D is shown in the graph. Scale bar: 50 μm. **P < 0.01. n = 5. (B) N1E-115 cells were transfected with Myc-NDPK-D and fractionated into cytosol and nuclear fractions. Each fraction was subjected to deacetylation assay as described in Fig 4. n = 4. (C) The HDAC inhibitor (TSA) seems to increase the acetylation level of NDPK-D in the cytosol fraction. n = 3. (D) Leptomycin B (LMB) treatment did not cause nuclear accumulation of NDPK-D. The cells transfected with Myc-tagged NDPK-D or HA-tagged zyxin (used as positive control) were cultured for 36 h, in the presence or absence of LMB during the last 6 h. The cells were stained with anti-Myc antibody for Myc-NDPK-D or anti-HA antibody for HA-zyxin. Scale bar: 50 μm. (E) SIRT1 causes nuclear accumulation of NDPK-D. N1E-115 cells were transfected with Myc-tagged WT NDPK-D and HA-tagged WT SIRT1 or deacetylase-deficient mutant SIRT1 (H363Y). After 36 h, cells were fixed and immunostained with anti-Myc and HA antibodies. NDPK-D localization was classified in N1E cells expressing both Myc-tagged NDPK-D and HA-tagged SIRT1. Scale bar: 20 μm. *P < 0.05, **P < 0.01. n = 4. (F) Acetylation-mimetic form of NDPK-D induced apoptosis. N1E-115 cells were transfected with Myc-tagged wild-type NDPK-D, the 3KR or the K45/72/91Q (3KQ) mutant. Cells were immunostained with anti-cleaved caspase-3 and anti-Myc antibodies. The frequencies of cleaved caspase-3-positive cells in the transfected cells were normalized to that of control. **P < 0.01. n = 5. (G) The treatment with SIRT1 inhibitor EX527 tended to increase the number of cleaved caspase-3-positive cells. N1E-115 cells were treated with or without EX527, and cultured for 48 h. Statistical analyses were performed using one-way ANOVA followed by Scheffe’s multiple comparison tests (A, D, E), or Welch’s t-test (B).

Since one of the acetylation target lysine residues are located within the RRK motif (R89–R90–K91), which is important for NDPK-D mitochondrial localization [37] (Fig 3B), we tested whether acetylation might affect the localization of NDPK-D. N1E-115 cells were transfected with plasmids encoding Myc-tagged NDPK-D WT, deacetylated mimetic form (3KR), or acetylated mimetic form (3KQ). While the NDPK-D WT was distributed in both the cytoplasm and the nucleus, 3KR mutants demonstrated a predominantly nuclear localization (Fig 5A). These observations suggest that deacetylation can lead to increased nuclear levels of NDPK-D. We also examined the acetylation levels of NDPK-D in the cytoplasm and the nucleus. The difference of NDPK-D acetylation between cytoplasm and nucleus was not significant (Fig 5B). To address the possibility that other molecules such as histone deacetylases (HDACs) regulate the acetylation level of NDPK-D, we treated the cells with HDAC inhibitor trichostatin A (TSA). The TSA treatment increased the acetylation level of NDPK-D in the cytosol fraction compared with nuclear fraction (Fig 5C). These results suggest that both SIRT1 and HDACs regulate the acetylation level of NDPK-D.

We next assessed the regulatory mechanism of NDPK-D localization. It has not been clearly defined whether NDPK-D has nuclear localization signals (NLSs) or nuclear export signals (NESs). To confirm the LMB activity, HA-tagged zyxin was used as a positive control [32]. We found that the treatment with LMB, an inhibitor of CRM1-mediated nuclear export, did not affect the subcellular localization of NDPK-D (Fig 5D). We further examined the effect of NDPK-D deacetylation by SIRT1 on the localization of NDPK-D. N1E-115 cells were transfected with plasmids encoding both Myc-tagged NDPK-D and HA-tagged SIRT1 WT or HA-tagged SIRT1 H363Y, a loss-of function mutant [22, 26]. Co-transfection of NDPK-D with SIRT1 WT but not SIRT1 H363Y increased nuclear accumulation of NDPK-D (Fig 5E). These results suggest that SIRT1 modulates deacetylation and nuclear localization of NDPK-D. To explore the physiological role of the acetylation level of NDPK-D, we examined cellular survival by using WT, 3KR, and 3KQ of NDPK-D. The number of cleaved caspase-3-positive cells increased in NDPK-D 3KQ transfected-N1E-115 cells (Fig 5F), whereas NDPK-D 3KR did not increase apoptosis. In addition, treatment of N1E-115 cells with Ex527, another SIRT1 inhibitor, tended to increase the number of cleaved caspase-3-positive cells (Fig 5G). These results suggest that increased acetylation level of NDPK-D induces cell death.

## Discussion

In this study, we demonstrated that NDPK-D knockdown induces caspase-3 activation, which in turn lead to apoptosis. Furthermore, SIRT1 deacetylates NDPK-D and mutation in the acetylated lysine residues of NDPK-D impaired mitochondrial localization of NDPK-D. The acetylation-mimetic form of NDPK-D causes apoptosis, suggesting that acetylation of NDPK-D regulates its ability to mediate apoptosis.

In addition to ATP generation and bioenergetic function, mitochondria also influence apoptosis. The alternative mitochondrial functions may be responsible for the pathogenesis of neurodegenerative diseases. NDPK-D, localized in the mitochondrial intermembrane space functions as a phosphotransferase using mitochondrial ATP and binds to anionic phospholipids such as cardiolipin. Cardiolipin shuttling between the inner and outer membrane is essential for the activation of apoptotic signaling [48–50]. Recent report demonstrated that NDPK-D mediates redistribution of intermembrane cardiolipin between inner membrane and outer membrane, sensitizing the cells to apoptosis [51]. Moreover, wild type NDPK-D overexpression in HeLa cells promotes cardiolipin transfer from inner membrane to outer membrane, and increases rotenone-induced apoptosis. However, overexpression of NDPK-D R90D mutant does not elicit these effects [51], indicating that NDPK-D mediates stress-induced apoptosis through cardiolipin translocation. Our results demonstrate that knockdown of NDPK-D induces apoptosis (Fig 2). We analyzed five control or NDPK-D siRNA-injected brains. To obtain these numbers of brains, we need seven pregnant mice for *in utero* electroporation. All NDPK-D siRNA-injected brains showed cell death (Fig 2G).

It has been shown that several mitochondrial proteins mediate dual functions, cellular energy homeostasis and apoptotic signaling [52–54]. For example, cytochrome c regulates the generation of mitochondrial transmembrane potential (ΔΨm), which is essential for ATP production. In contrast, cytochrome c release to the cytosol induces caspase activation, causing apoptosis [55, 56]. Considering these observations, our results suggest that NDPK-D can be involved in both life and death decisions.

We also assessed the mechanism of cell death mediated by NDPK-D. We used N1E-115 cells to explore the mechanism of cell death in order to reduce the number of experimental animals. This is in accordance with the National Centre for the Replacement, Refinement and Reduction of Animals in Research (London, UK). NDPK-D 3KQ but not 3KR induced cell death in N1E-115 cells, suggesting that increased acetylation level of NDPK-D induces apoptosis (Fig 5F).

We previously reported that SIRT1 is preferentially expressed in developing mouse brain [32]. In this study, we show that NDPK-D is predominantly expressed at embryonic stage (Fig 1A). Moreover, both proteins are highly expressed in the ventricular zone of embryonic mouse brain (Fig 1B) [39, 57]. These findings suggest that NDPK-D execute some biological functions in the developing brain.

The present study identifies and confirms that overexpressed NDPK-D and overexpressed or endogenous SIRT1 have bound to each other. Even though NDPK-D has a specific mitochondrial targeting sequence, overexpressed NDPK-D can localize to the nucleus in addition to the cytoplasm (Fig 3B). Because transfected SIRT1 showed predominant nuclear localization, in COS-7 cells, NDPK-D and SIRT1 may interact in the nucleus. Furthermore, SIRT1 deacetylates NDPK-D in N1E-115 cells (Fig 4A–4D) and non-acetylated NDPK-D (3KR) mutant accumulates in the nucleus fraction (Fig 5A), suggesting that SIRT1 could modulate the role of NDPK-D in the nucleus through deacetylation. It is also possible that SIRT1 modulates NDPK-D localization. SIRT1 has two NLSs and NESs, which allow it to translocate between the cytosol and the nucleus [58]. However, a NLS has not been identified in NDPK-D. There was no significant difference in the acetylation levels of NDPK-D between nuclear and cytosol fractions (Fig 5B). The reason why we did not observe difference may be due to the partial deacetylation of NDPK-D by SIRT1. Our results showing that inhibition of HDACs by TSA seems to increase the acetylation level of NDPK-D in the cytoplasm support this possibility (Fig 5C). While this study has clearly demonstrated the subcellular localization of NDPK-D, we need to determine the biological function of the NDPK-D translocation between the cytosol and the nucleus.

Both SIRT1 and NDPK-D function in cell survival. SIRT1 can protect neurons from oxidative stress [59] and neurotoxic insults in several models for Alzheimer’s disease, ALS [15], and Wallerian degeneration [14]. Furthermore, SIRT1 is upregulated during progressive neurodegeneration [15]. This study indicates that NDPK-D is required for cellular survival in N1E-115 cells and embryonic mouse cortex (Fig 2). Although both NDPK-D siRNA #1 and #3 induced caspase-3 activation 72 h after the transfection (Fig 2F), NDPK-D siRNA#1, but not #3, induced apoptosis 36 h after the transfection (Fig 2E). NDPK-D siRNA #3 seems to need a longer time to maximize the effect of cell death than siRNA #1. Further, SIRT1 increases nuclear accumulation of NDPK-D (Fig 5). These observations raise the possibility that nuclear translocation triggers the deacetylation of NDPK-D by SIRT1, thereby protecting the cells from apoptosis.

In summary, NDPK-D plays a crucial role in cell survival. SIRT1 binds to NDPK-D, and mutation in NDPK-D lysine residues promotes its nuclear accumulation. Further, the acetylation-mimetic mutant of NDPK-D induces apoptosis. Taken together, our results suggest that SIRT1 regulates the localization of NDPK-D and cell survival via deacetylation.

## Supporting Information

S1 Checklist NC3Rs ARRIVE Guidelines Checklist. (PDF)

S1 Fig Three lysine residues in human NDPK-D. The predicted three acetylation sites Lys-45, Lys-72, and Lys-91 were indicated as bold letter.
(TIF)
