# Supplementary figures and images for: Acetylation of NDPK-D Regulates Its Subcellular Localization and Cell Survival
Source: PLoS One. 2015 Oct 1;10(10):e0139616. doi: 10.1371/journal.pone.0139616 (PMC4591271; doi:10.1371/journal.pone.0139616)

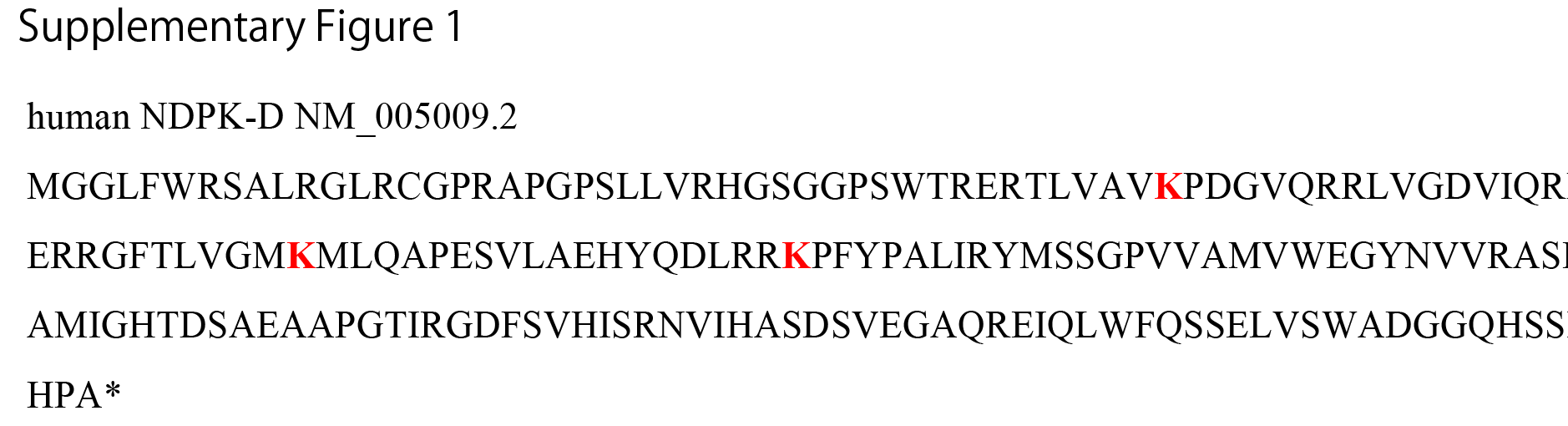

Supplement: S1 Fig — The predicted three acetylation sites Lys-45, Lys-72, and Lys-91 were indicated as bold letter. (TIF) [file pone.0139616.s002.tif]
